# Angiotensin-I-Converting Enzyme (ACE)-Inhibitory Peptides from the Collagens of Monkfish (*Lophius litulon*) Swim Bladders: Isolation, Characterization, Molecular Docking Analysis and Activity Evaluation

**DOI:** 10.3390/md21100516

**Published:** 2023-09-28

**Authors:** Yu-Dong Hu, Qing-Hao Xi, Jing Kong, Yu-Qin Zhao, Chang-Feng Chi, Bin Wang

**Affiliations:** 1 Zhejiang Provincial Engineering Technology Research Center of Marine Biomedical Products, School of Food and Pharmacy, Zhejiang Ocean University, Zhoushan 316022, China; 2 National and Provincial Joint Laboratory of Exploration and Utilization of Marine Aquatic Genetic Resources, National Engineering Research Center of Marine Facilities Aquaculture, School of Marine Science and Technology, Zhejiang Ocean University, Zhoushan 316022, China

**Keywords:** monkfish (*Lophius litulon*), swim bladder, collagen, angiotensin-I-converting enzyme (ACE), peptides, molecular docking analysis

## Abstract

The objective of this study was to isolate and characterize collagen and angiotensin-I-converting enzyme (ACE)-inhibitory (ACEi) peptides from the swim bladders of monkfish (*Lophius litulon*). Therefore, acid-soluble collagen (ASC-M) and pepsin-soluble collagen (PSC-M) with yields of 4.27 ± 0.22% and 9.54 ± 0.51%, respectively, were extracted from monkfish swim bladders using acid and enzyme methods. The ASC-M and PSC-M contained Gly (325.2 and 314.9 residues/1000 residues, respectively) as the major amino acid, but they had low imino acid content (192.5 and 188.6 residues/1000 residues, respectively) in comparison with collagen from calf skins (CSC) (216.6 residues/1000 residues). The sodium dodecyl sulfate–polyacrylamide gel electrophoresis (SDS-PAGE) patterns and ultraviolet (UV) absorption spectrums of ASC-M and PSC-M illustrated that they were mainly composed of type I collagen. Subsequently, three ACEi peptides were isolated from a PSC-M hydrolysate prepared via a double-enzyme system (alcalase + neutrase) and identified as SEGPK (MHP6), FDGPY (MHP7) and SPGPW (MHP9), with molecular weights of 516.5, 597.6 and 542.6 Da, respectively. SEGPK, FDGPY and SPGPW displayed remarkable anti-ACE activity, with IC_50_ values of 0.63, 0.94 and 0.71 mg/mL, respectively. Additionally, a molecular docking assay demonstrated that the affinities of SEGPK, FDGPY and SPGPW with ACE were −7.3, −10.9 and −9.4 kcal/mol, respectively. The remarkable ACEi activity of SEGPK, FDGPY and SPGPW was due to their connection with the active pockets and/or sites of ACE via hydrogen bonding, hydrophobic interaction and electrostatic force. Moreover, SEGPK, FDGPY and SPGPW could protect HUVECs by controlling levels of nitric oxide (NO) and endothelin-1 (ET-1). Therefore, this work provides an effective means for the preparation of collagens and novel ACEi peptides from monkfish swim bladders, and the prepared ACEi peptides, including SEGPK, FDGPY and SPGPW, could serve as natural functional components in the development of health care products to control hypertension.

## 1. Introduction

The output of global fish and fish-related supplements has reached approximately 179 million tons, in which the proportion from marine fishery accounts for approximately 84.4 million tons [1,2]. During the manufacturing process, approximately 30–50% of these fish are processed into by-products, which leads to major economic and environmental problems [3,4]. Therefore, some functional components, such as collagen/gelatin, astaxanthin, polysaccharides, unsaturated fatty acids, protease and active peptides, are prepared from fish by-products using some bioprocessing technologies to produce high-value functional goods [5,6,7].

Collagen with a triple-helix structure is one of the most common types of protein (accounting for about 30% of the body’s proteins) and serves as the primary building block of the skin, cartilage, blood vessels, muscles, ligaments, bones, internal organs and connective tissues of the human body [8,9]. As of the present, researchers have purified and identified twenty-nine types of collagens (types I-XXIX), which differ depending on the way the molecules, added cell components and used tissues are assembled. In addition, a large number of collagens originating from mammalian by-products have been widely used in foods, cosmetics, nourishment, photography and pharmaceutical/biomedical materials [10,11]. However, mammalian collagens and their derivatives cause concern and anxiety among consumers because of viral infectious diseases and ethnic dietary restrictions; therefore, collagen producers actively develop alternative products [9,12]. Due to the unique existence of marine life, collagen from marine organisms does not have the same infectious disease problems as land-based collagen and can be widely consumed in Muslim and Hindu countries [13]. Moreover, the demand for marine collagen is gradually increasing among industrialists due to its unique physicochemical properties, abundant resources and safe reliability [3,14]. Therefore, marine collagens have been widely extracted from different fish by-products [13,15].

Due to their low proportion of imino acids, the thermal and structural stability of marine collagens are inferior to those of mammal collagens, and they are more suitable for degradation by proteases to produce bioactive collagen hydrolysates and peptides [16]. Compared with regular collagens, hydrolyzed collagens have high bioavailability because they are easily dissolved in water and absorbed into the bloodstream. Moreover, hydrolyzed marine collagens or peptides present multifarious biological functions, such as antioxidant [17], UV damage resistance [18], iron-chelating [19], wound-healing [20,21], osteoporosis-preventing [22], mucosa-pairing [23], antiaging [24], antifreezing [25], immunity-enhancing [26], hepatoprotective [27] and ACE-inhibiting (ACEi) activity [28]. Therefore, the preparation of bioactive peptides using marine-derived collagen and gelatin has been widely taken into consideration because of their significant functions and promising applications [21].

As a common clinical disease, hypertension can affect the health of the body’s arteries and becomes an enormous and latent risk factor for cardiovascular diseases (CVDs), atherosclerosis, heart failure, etc. [29,30]. However, the World Health Organization (WHO) reports that the number of people with hypertension will increase from 1.28 billion to 1.56 billion by 2030 [31]. ACE can modify angiotensin (Ang) I to active Ang II to up-regulate blood pressure by inactivating the vasodilator bradykinin. In consequence, the inhibition of ACE activity is an important way to control systemic high blood pressure [29,30]. Chemosynthetic ACEi drugs, such as quinapril, captopril (Cap), lisinopril and ramipril, have been used to reduce hypertension in clinical settings, but side effects seriously affect the application of these ACEi drugs [32]. The search for safer ACEi drugs from animals and plants can offer a viable alternative to chemosynthetic ACEi drugs for lowering high blood pressure [33,34,35]. Therefore, the preparation of ACEi peptides from fish and fish by-products has become a focal point for researchers, and some ACEi peptides have been prepared from different marine organisms, such as *Pacific cod* [36], *Takifugu bimaculatus* [28], *Skipjack tuna* [37,38], squilla [39], *Alaska pollack* [40], *hybrid groupers* [41], *miiuy croakers* [42], *Kuruma shrimp* [31], *Cobia* [43] and *Antarctic krill* [44].

The monkfish (*Lophius litulon*) belongs to the genus Lophius and is captured mainly in northeast China and Asia, with the production of approximately 2 × 10^5^ tons/year [45]. As of the present, multifarious peptides have been purified from monkfish and its by-products and have displayed significant physiological and biological activity. For example, peptides from monkfish muscle could effectively ameliorate kidney damage in mice caused by a high-fat diet (HFD) by improving antioxidant activity, decreasing inflammatory cytokine production and regulating intestinal dysbiosis [46]. Antioxidant peptides from swim bladders as monkfish by-products could improve the treatment of nonalcoholic fatty liver disease (NAFLD) by controlling oxidative stress and lipid accumulation via regulating AMP-activated protein kinase (AMPK) and nuclear factor erythroid 2-related factor 2 (Nrf2) signal pathways [47,48]. Low-molecular-weight (MW) peptides from roes as monkfish by-products could significantly promote immune response in immunosuppressed mice [49]. Antioxidant oligopeptides from monkfish muscle could enhance the protective ability of HepG2 cells to avoid H_2_O_2_-induced damage [50]. Collagen peptides from monkfish skins could ameliorate renal injury in HFD-induced mice by controlling the Nrf2 and NOD-like receptor thermal protein domain associated protein 3 (NLRP3) signal pathways [51]. Low-MW peptides from monkfish muscle and liver present an antifatigue effect [42,43,44,45,46,47,48,49,50,51,52,53]. The swim bladder is a by-product of the processing of monkfish, but it is often discarded as waste, resulting in resource waste and environmental pollution. The question of how to use swim bladders efficiently is of great significance. Thus, the aim of this research is to isolate and characterize collagens (ASC-M and PSC-M) from monkfish (*L. litulon*) swim bladders and their derived ACEi peptides. In addition, the ACEi activity and mechanism of the prepared peptides are also investigated.

## 2. Results and Discussion

### 2.1. Preparation and Characterization of Collagen from Swim Bladders of Monkfish (L. litulon)

#### 2.1.1. Proximate Composition Analysis

The chemical compositions of the monkfish (*L. litulon*) swim bladders, ASC-M and PSC-M are presented in Table 1. The protein content in monkfish swim bladders was 20.06 ± 1.09%, which was higher than that in the swim bladders of *Miichthys miiuy* (19.17 ± 0.15%) [11], catla (*Catla catla*) (14.28%) [54] and yellowfin tuna (12.09%) [55]. Furthermore, the protein content on the dry weight basis of ASC-M and PSC-M was 93.68 ± 3.51% and 95.87 ± 2.89%, respectively, which was significantly higher than that in monkfish swim bladders (*p* < 0.05). These results illustrated that noncollagenous proteins, minerals, sugars, etc. were effectively cleared from monkfish swim bladders through the presented extraction method.

As shown in Table 1, the yield of ASC-M was 4.27 ± 0.22%, which is significantly higher than those of ACS from the swim bladders of miiuy croakers (1.33%) [11] and yellowfin tuna (1.07%) [55]. In addition, the yield of PSC-M was 9.54 ± 0.51% and was 2.23-fold that of ASC-M. These data confirmed that more cross-linked collagens exist in monkfish swim bladders. Therefore, the telopeptide region in the collagens was specifically cleaved by pepsin, which made the collagen structure smaller and was more conducive to its extraction from the fibril matrix [11].

#### 2.1.2. Amino Acid Analysis of ASC-M and PSC-M

Table 2 shows that the amino acid compositions of ASC-M and PSC-M had a similar pattern. ASC-M and PSC-M were rich in Gly (325.2 and 314.9 residues/1000 residues, respectively), followed by Pro (105.2 and 102.9 residues/1000 residues, respectively), Ala (99.3 and 97.6 residues/1000 residues, respectively) and Glu/Gln (88.6 and 87.1 residues/1000 residues, respectively). Gly accounts for about 1/3 of the total amino acids, which is consistent with the literature reports that all types of collagens are typically characterized by a repetitive domain of tripeptides (Gly-X-Y) involved in the formation of the triple-helix structure [11,55]. In addition, Gly is the key amino acid in the three helix chains forming the superhelix structure of collagen.

The imino acid content of ASC-M and PSC-M was 192.5 and 188.6 residues/1000 residues, respectively (Table 2), which was similar to that of collagens from the swim bladders of sea bass (194 residues/1000 residues) [56] and *Megalonibea fusca* (195 and 199 residues/1000 residues) [57], but significantly lower than that of pig skin collagen and calf skin collagen (CSC) (216.6 and 220 residues/1000 residues, respectively) [58]. Imino acids are a critical factor in maintaining the structural and thermal integrity of collagens. The pyrrolidine rings of Hyp and Pro can restrain changes in the secondary structure of a polypeptide chain; therefore, the stability of the triple-helix structure is improved. In addition, the hydroxyl group of Hyp can form interchain hydrogen bonds to stabilize the collagen’s triple-stranded helix [9,59]. Therefore, the lower ratios of imino acids in ASC-M and PSC-M resulted in their thermal and structural stability being weaker than that of collagens from calf and pig skins.

#### 2.1.3. Electrophoretic Patterns of ASC-M and PSC-M

**Figure 1:**
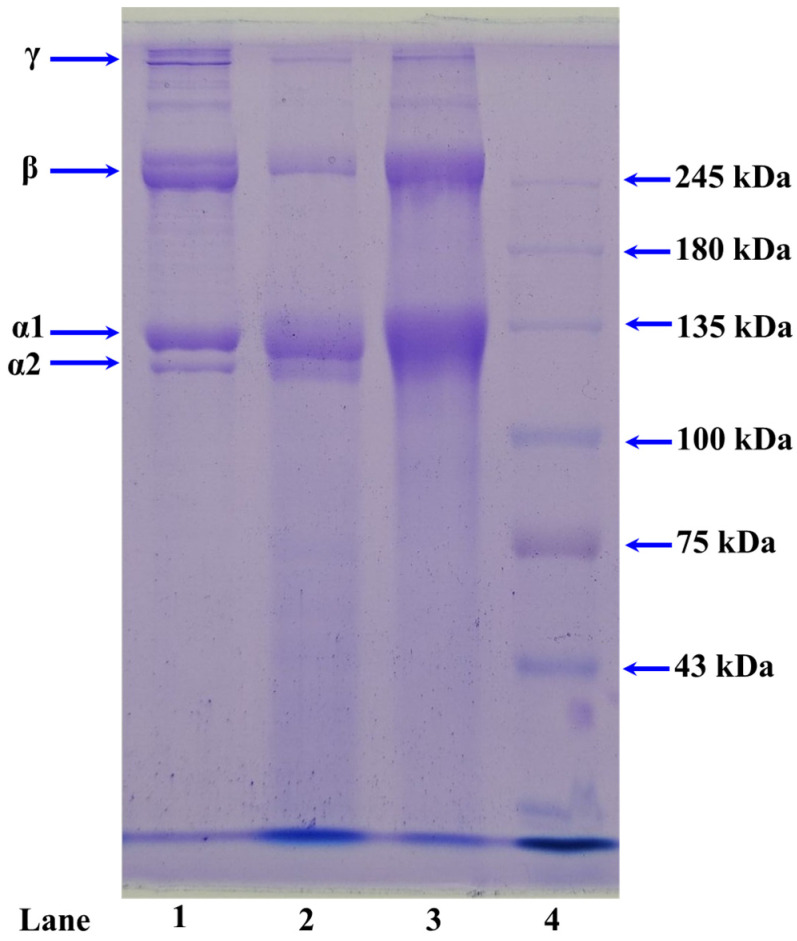
SDS-PAGE patterns of ASC-M and PSC-M from swim bladders of monkfish (*L. litulon*). Lane 1: CSC (type I collagen); lane 2: PSC-M; lane 3: ASC-M; lane 4: marker protein.

Figure 1 shows that ASC-M and PSC-M showed similar SDS-PAGE patterns. Two α-chains (α1 and α2, with MWs ranging from 110 to 120 kDa) were the major constituents of ASC-M (lane 3) and PSC-M (lane 2), which was consistent with the protein pattern of type I collagen ([α1]_2_α2) in CSC (lane 1). These results suggested that type I collagen was the main component of ASC-M and PSC-M. According to the previous literature, we found that collagens from the swim bladders of *M. fusca* [57], *Lates calcarifer* [56], *Ctenopharyngodon idella* [60], Gulf corvina [61], miiuy croakers [11], *Totoaba macdonaldi* [62] and yellowfin tuna [55] have similar SDS-PAGE patterns to ASC-M and PSC-M. In addition, the SDS-PAGE patterns showed that ASC-M and PSC-M contained more low-MW bands compared with CSC, which suggested that the high-MW components, including the β-chain and γ-chain, were cleaved into low-MW peptides in the extraction process.

#### 2.1.4. Ultraviolet (UV) Absorption Spectrums of ASC-M and PSC-M

**Figure 2:**
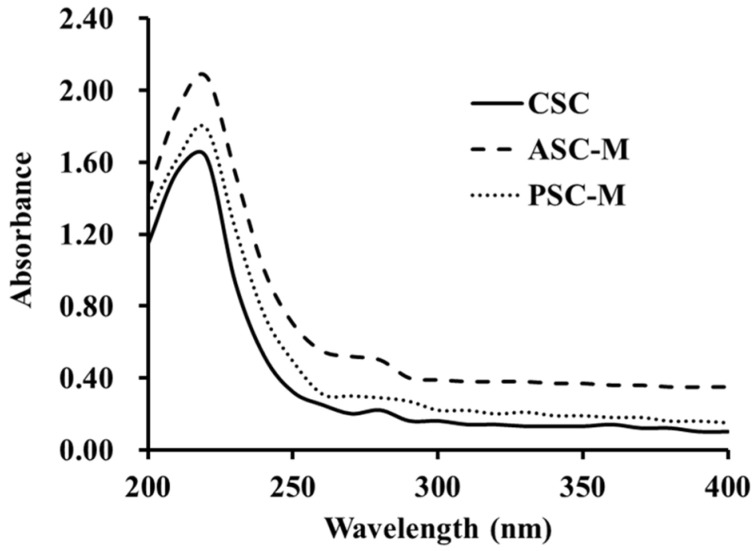
UV spectrums of ASC-M and PSC-M from swim bladders of monkfish (*L. litulon*).

Figure 2 shows that ASC-M, PSC-M and CSC had similar UV absorption peaks, ranging from 225 to 230 nm, which was similar to reports for collagens extracted from the swim bladders of Gulf corvina [61], *M. miiuy* [11] and *M. fusca* [57]. This maximum absorption at 220–240 is associated with the groups of carbonyl (C=O), carboxyl (–COOH) and amido bonds (CONH_2_) in the polypeptide chains of ASC-M and PSC-M. Additionally, a small absorption peak was detected at approximately 280 nm, which illustrated that ASC-M and PSC-M were composed of low aromatic amino acid content, which agrees with the data in Table 2.

### 2.2. Preparation of ACEi Peptides from Collagen Hydrolysates of Monkfish Swim Bladders

#### 2.2.1. Preparation of Collagen Hydrolysates of Monkfish Swim Bladders

**Figure 3:**
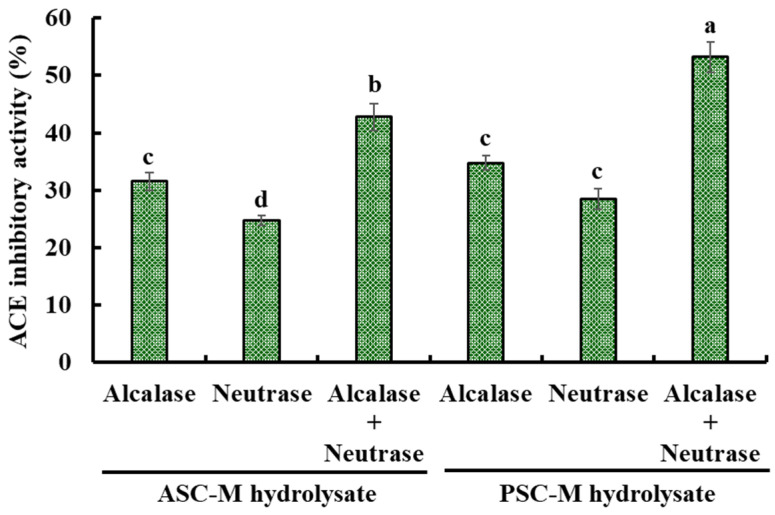
The ACEi activity of hydrolysates from ASC-M and PSC-M from monkfish (*L. litulon*). ^a–d^ Values with same letters indicate no significant difference (*p* > 0.05).

ASC-M and PSC-M were separately hydrolyzed by alcalase, neutrase and a double-enzyme system (alcalase + neutrase), and the ACEi activity of the generated hydrolysates at 2.5 mg/mL is depicted in Figure 3. The ACEi activity of the PSC-M hydrolysate prepared via a double-enzyme system (alcalase + neutrase) was 53.22 ± 2.63%, which was significantly higher than the ACEi activity of the other collagen hydrolysates (*p* < 0.05). In addition, Figure 3 shows that the ACEi activity of the PSC-M hydrolysates was better than that of the ASC-M hydrolysates. Therefore, the PSC-M hydrolysate prepared via a double-enzyme system (alcalase + neutrase) was named PSC-MH and chosen for follow-up experiments.

#### 2.2.2. Preparation of ACEi Peptides from PSC-MHs

**Figure 4:**
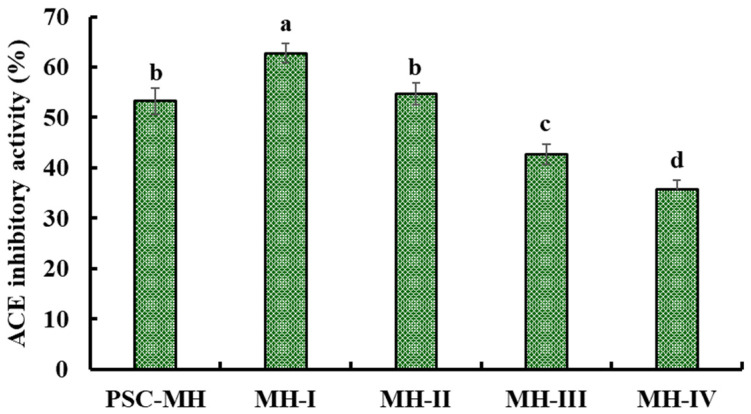
The ACEi activity of fractions from PSC-MH prepared via ultrafiltration membranes. ^a–d^ Values with same letters indicate no significant difference (*p* > 0.05).

Through three ultrafiltration membranes, four fractions, namely MH-I, MH-II, MH-III and MH-IV, were separated from PSC-MH. Moreover, the activity of MH-I was 62.75 ± 1.89% at 2.5 mg/mL, which was significantly higher than those of PSC-MH (53.22 ± 2.63%), MH-II (54.73 ± 2.19%), MH-III (42.67 ± 2.03%) and MH-IV (35.69 ± 1.87%) (*p* < 0.05) (Figure 4). Ultrafiltration is a popular technique for concentrating target fractions from protein hydrolysates according to their molecular size [6]. After ultrafiltration, MH-I enriched more low-MW peptides, which have a higher chance of combining with ACE [63]. Similarly, low-MW peptide fractions from hydrolysates of *Takifugu bimaculatus* [28], tuna dark muscle [37], *Mytilus edulis* [30,32], hybrid groupers [41], Pacific cod [36] and miiuy croakers [42] also showed high ACEi activity. Therefore, MH-I was subsequently analyzed using chromatographic methods.

**Figure 5:**
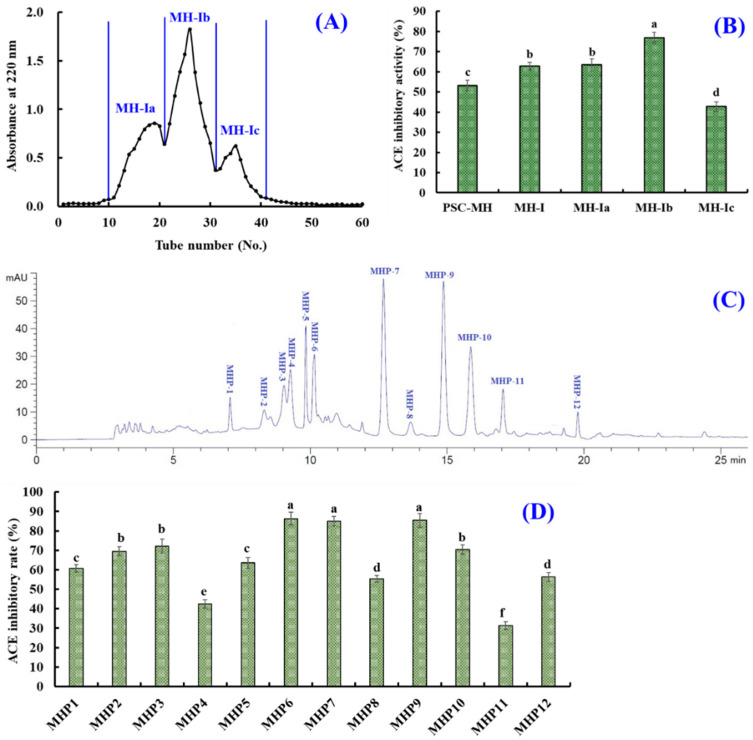
Separation of MH-I and ACEi activity of isolated peptides. (**A**) Chromatogram of MH-I purified with Sephadex G-25 column. (**B**) ACEi activity of fractions (MH-Ia to MH-Ic) from MH-I at 2.5 mg/mL. (**C**) HPLC profile of MH-Ib at 220 nm. (**D**) ACEi activity of peptides (MHP-1 to MHP-12) from MH-Ib at 1.5 mg/mL. ^a–f^ Values with same letters indicate no significant difference (*p* > 0.05).

MW is one of the key elements to be focused during the purification process of bioactive peptides [10,64]. Thus, MH-I was further divided into three components (MH-Ia, MH-1b and MH-Ic) with a Sephadex G-25 column (Figure 5A). The ACEi activity of MH-1b was 76.81 ± 2.76%, which was significantly higher than those of PSC-MH (53.22 ± 2.63%), MH-I (62.75 ± 1.89%), MH-Ia (63.39 ± 3.06%) and MH-Ic (42.77 ± 2.38) (*p* < 0.05) (Figure 5B). Gel filtration is an efficient technology for separating bioactive substances with different MWs and is widely used to purify ACEi peptides from different hydrolysates of marine proteins, such as *Skipjack tuna* roes [34], *Ulva prolifera* [65], *Kuruma shrimp* [66], *Ruditapes philippinarum* [67], pearl oyster [68], squilla [39], hybrid groupers [41], etc. Although MH-1b presented the highest ACEi activity, its MW was not the lowest among the three peptide components. This finding illustrated that other properties besides MW, such as composition and sequence of amino acids, hydrophilicity/hydrophobicity and spatial structure, also greatly influence the ACEi activity of peptides.

MH-1b was finally separated using RP-HPLC (Figure 5C). According to the elution chromatogram of MH-1b at 220 nm, twelve ACEi peptides with retention times (RTs) of 7.03 (MHP1), 8.21 (MHP2), 8.99 (MHP3), 9.17 (MHP4), 9.76 (MHP5), 10.08 (MHP6), 12.68 (MHP7), 13.73 (MHP8), 14.91 (MHP9), 15.92 (MHP10), 17.03 (MHP11) and 19.81 min (MHP12) were isolated and collected. The ACEi activity values of MHP6, MHP7 and MHP9 at 1.5 mg/mL were 86.34 ± 3.25%, 84.99 ± 2.34% and 85.39 ± 3.46%, respectively, which were significantly higher than the activity values of the other nine prepared ACEi peptides (*p* < 0.05) (Figure 5D).

RP-HPLC is an extremely efficient technology for isolating ACEi peptides according to their RT, and the RT of separated ACEi peptides can be modulated by adjusting the ratio of polar solvent (such as trifluoroacetic acid (TFA), methanol, ethyl alcohol and acetonitrile) in the mobile phase [30,32,64]. Therefore, ACEi peptides have been separated using RP-HPLC from protein hydrolysates of *T. bimaculatus* [28], *R. philippinarum* [67], *Skipjack tuna* roes [31], *M. edulis* [30], *Kuruma shrimp* [66], pearl oyster [68], *Pyropia pseudolinearis* [69], *squilla* [39], *hybrid groupers* [41], *Arthrospira platensis* [70], etc.

### 2.3. Sequences and MW Determination of MHP6, MHP7 and MHP9

**Figure 6:**
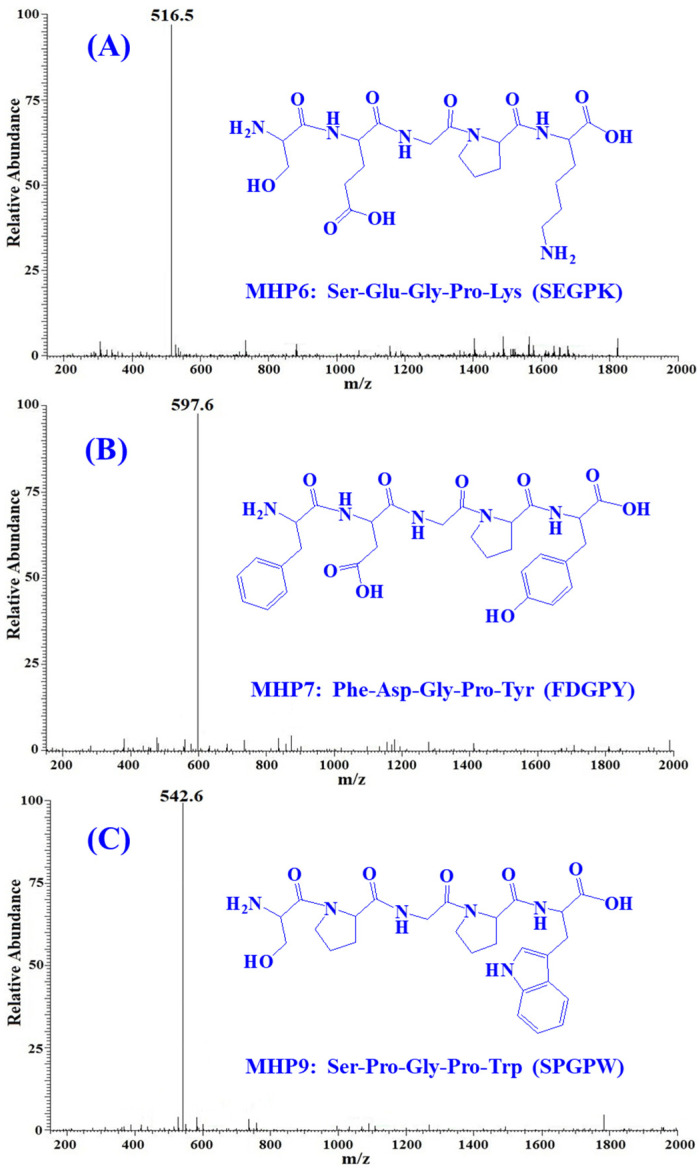
Mass spectrums of three isolated ACEi peptides from PSC-MH. (**A**) MHP6 (SEGPK); (**B**) MHP7 (FDGPY); (**C**) MHP9 (SPGPW).

MHP6, MHP7 and MHP9 were analyzed using a protein/peptide sequencer and their sequences were identified as Ser-Glu-Gly-Pro-Lys (SEGPK), Phe-Asp-Gly-Pro-Tyr (FDGPY) and Ser-Pro-Gly-Leu-Trp (SPGPW), respectively. The MWs of MHP6, MHP7 and MHP9 were measured as 516.5, 597.6 and 542.6 Da, respectively, and the data agreed with the theoretical MWs of MHP6, MHP7 and MHP9 (Figure 6, Table 3).

### 2.4. IC_50_ Values and Molecular Docking Analysis of MHP6, MHP7 and MHP9

**Figure 7:**
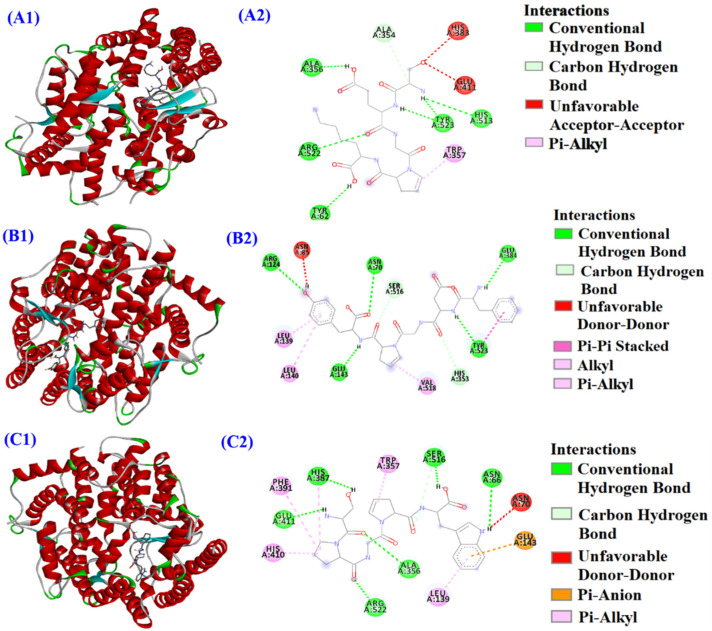
Molecular docking results of MHP6 (SEGPK) (**A**), MHP7 (FDGPY) (**B**) and MHP9 (SPGPW) (**C**) with ACE. (**A1**,**A2**): Two- and three-dimensional details of ACE and MHP6 (SEGPK) interaction; (**B1**,**B2**): Two- and three-dimensional details of ACE and MHP7 (FDGPY) interaction; (**C1**,**C2**): Two- and three-dimensional details of ACE and MHP9 (SPGPW) interaction.

As shown in Table 3, the IC_50_ value of MHP6 on ACE was 0.63 mg/mL, which was lower than those of MHP7 and MHP9 (0.94 and 0.71 mg/mL, respectively). To illustrate the ACEi mechanism of MHP6, MHP7 and MHP9, a molecular docking assay was conducted (Figure 7), and the affinities of MHP6, MHP7 and MHP9 with ACE were −7.3, −10.9 and −9.4 kcal/mol, respectively, which were similar to those of ACEi peptides from miiuy croakers (IKSW: −9.3 kcal/mol) [31], *M. edulis* (LSFR: −8.5 kcal/mol) [30], rice bran (YSK: −7.9 kcal/mol) [71], tuna milts (YEGDP: −8.8 kcal/mol) [32] and soybean (LVLL: −8.6 kcal/mol) [72].

Figure 7A proves that MHP6 (SEGPK) could combine with the Tyr62, Arg522, Ala356, Ala354 (S1), Tyr523 (S1) and His513 (S2) residues of ACE via hydrogen bonds. In addition, MHP6 (SEGPK) had a hydrophobic effect on the Trp357 residue of ACE. Figure 7B indicates that MHP7 (FDGPY) could form hydrogen bonds with the Arg124, Asn70, Glu143, Glu384 (S1), Try523 (S1), His516 and His353 (S2) residues of ACE. Aside from this, MHP7 (FDGPY) could establish interactions with the Tyr523 (S1), Leu139, Leu140 and Val518 residues of ACE via the hydrophobic effect. Figure 7C indicates that MHP9 (SPGPW) could form hydrogen bonds with the His387, Glu411, Arg522, Ala356, Ser516 and Asn66 residues of ACE. Besides this, MHP9 (SPGPW) could interact with ACE’s Phe391, His410, Leu139 and Trp357 residues via the hydrophobic effect, and could make contact with the Glu143 residue of ACE through electrostatic force. The ACEi activity of MHP6 and MHP7 is related to the combination with ACE’s S1 and S2 pockets, and their inhibitory effect on ACE is a competitive inhibition mode. However, MHP9 has a noncompetitive mode of inhibition because it does not bind with the S1 or S2 pockets.

### 2.5. Effects of MHP6, MHP7 and MHP9 on HUVECs

#### 2.5.1. Effects of MHP6, MHP7 and MHP9 on Viability of HUVECs

**Figure 8:**
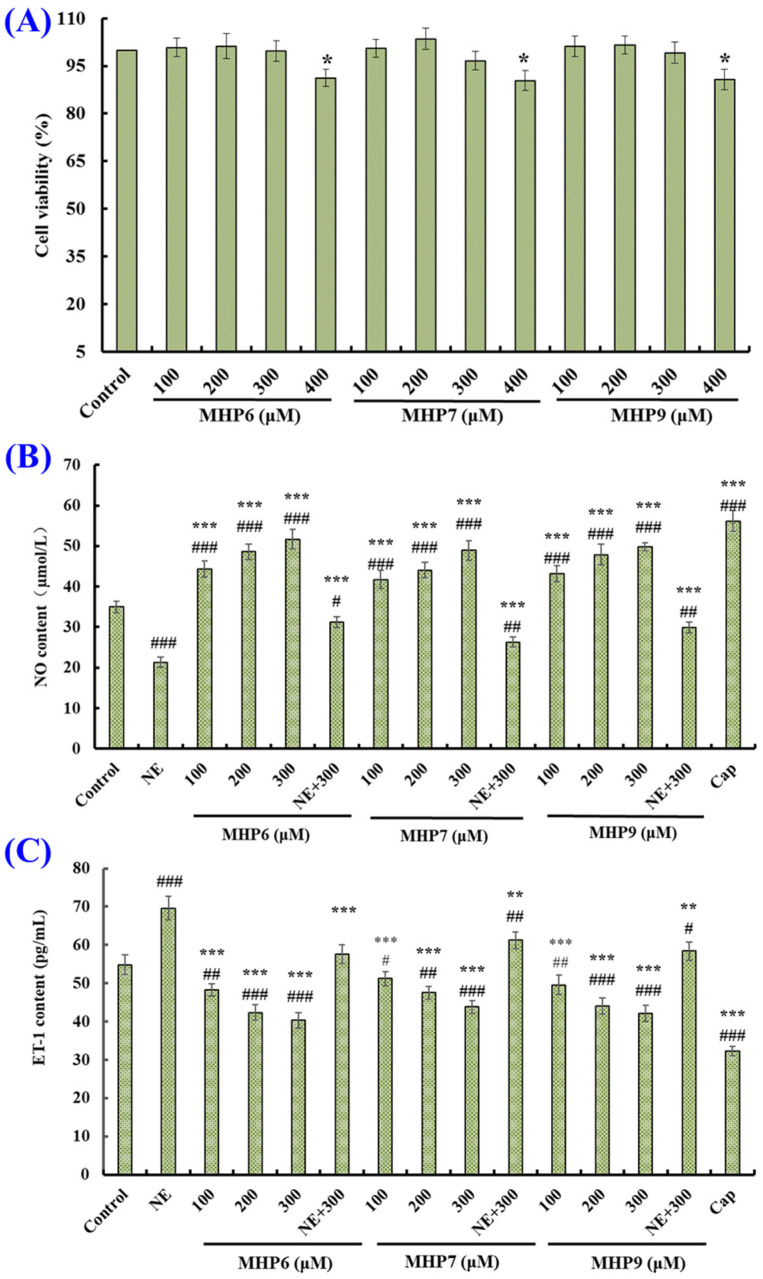
Effects of MHP6, MHP7 and MHP9 on viability (**A**), NO level (**B**) and ET-1 production (**C**) of HUVECs. Compared with control group: ^#^ *p* < 0.05, ^##^ *p* < 0.01 and ^###^ *p* < 0.001; compared with NE group: * *p* < 0.05, ** *p* < 0.01 and *** *p* < 0.001.

The viability of HUVECs treated with MHP6, MHP7 and MHP9 at 100–400 μM are depicted in Figure 8A. The cell viability values of the MHP6, MHP7 and MHP9 groups at 400 μM were 91.27 ± 2.64%, 90.39 ± 3.19% and 91.76 ± 3.22%, respectively, which were significantly lower than the cell viability values of the control and other concentration groups of MHP6, MHP7 and MHP9 (*p* < 0.05). These results indicated that MHP6, MHP7 and MHP9 at 400 μM might lead to some adverse harm to the function of HUVECs. Therefore, MHP6, MHP7 and MHP9 at 100, 200 and 300 μM were set for the follow-up experiments.

#### 2.5.2. Effects of MHP6, MHP7 and MHP9 on Production of NO and ET-1

Figure 8B shows that the NO level was markedly increased from 35.01 ± 1.38 µM to 56.18 ± 2.58 µM after HUVECs were treated by Cap, but significantly decreased to 21.34 ± 1.24 µM after HUVECs were treated by NE (*p* < 0.001). Moreover, the NO content in HUVECs was significantly increased by treatment with MHP6, MHP7 and MHP9. At 300 μM, the NO production levels in HUVECs incubated with MHP6, MHP7 and MHP9 increased to 51.68 ± 2.38, 48.91 ± 2.35 and 49.83 ± 0.96 µM, respectively. Furthermore, the NO content reversely descended by NE could be separately raised to 31.24 ± 1.29, 26.31 ± 1.22 and 29.87 ± 1.34 µM after being dealt with using MHP6, MHP7 and MHP9, respectively, at 300 μM (*p* < 0.001). The present findings manifest that MHP6, MHP7 and MHP9 could significantly promote the NO generation in HUVECs and make up for the loss of NO content caused by NE.

As depicted in Figure 8C, Cap at 1.0 μM could significantly reduce the ET-1 level in HUVECs from 54.87 ± 2.57 to 32.25 ± 1.26 pg/mL. Conversely, NE at 0.5 μM could increase the ET-1 content to 69.58 ± 3.11 pg/mL (*p* < 0.001). Furthermore, MHP6, MHP7 and MHP9 could significantly reduce the ET-1 content in HUVECs at 100–300 μM (*p* < 0.001), and the ET-1 content in MHP6, MHP7 and MHP9 groups dropped to 40.35 ± 1.96, 43.87 ± 1.73 and 42.18 ± 2.09 pg/mL, respectively, at 300 μM. Moreover, the increased ET-1 content caused by NE was partially reduced through MHP6, MHP7 and MHP9 treatment and lowered to 57.62 ± 1.95, 61.23 ± 1.89 and 58.44 ± 2.34 pg/mL, respectively, at 300 μM (*p* < 0.001). These findings indicated that MHP6, MHP7 and MHP9 had great ability to reduce the ET-1 secretion in HUVECs.

NO is the most potent vascular endothelium-derived vasodilator and performs a vital function in preventing CVDs [31]. In addition, ET-1 is a known vasoconstriction factor, similar to Ang II, that can result in endothelial dysfunction associated with high blood pressure and atherosclerosis [30]. WGESF and WSPGF from tuna roes could significantly bring down ET-1 secretion and improve the NO generation in HUVECs, but also reverse negative influences of NE on the levels of NO and ET-1 [31]. IVTNWDDMEK and VGPAGPRG could regulate NO and ET-1 production in a concentration-dependent manner and positively regulate the expression levels of Nrf2 and heme oxygenase-1 (HO-1) proteins to protect HUVECs against oxidative damage [73]. ACEi peptides from *T. bimaculatus* skins (FNLRMQ) [28], tuna milts (ICY, LSFR and IYSP) [32], yak milk casein (KYIPIQ) [74], *M. edulis* (YEGDP and WF) [30], *Isochrysis zhanjiangensis* (EMFGTSSET) [75] and miiuy croaker swim bladders (SHGEY and SPYGF) [42] showed similar regulatory function to that of the functional factors that protect HUVECs and oxidation-damaged HUVECs. The present results indicate that MHP6, MHP7 and MHP9 had a great ability to regulate the secretion of NO and ET-1 in HUVECs, which has positive effects on the protection of vascular endothelial cells and the treatment of high blood pressure.

## 3. Materials and Methods

### 3.1. Chemicals and Reagents

Monkfish swim bladders were provided by Zhejiang Hailisheng Group Co., Ltd. (Zhoushan, China). High-MW markers were bought from the Shanghai Institute of Biochemistry (Shanghai, China). Porcine pepsin and CSC were obtained from Sigma-Aldrich (Shanghai, China) Trading Co., Ltd. (Shanghai, China). Human umbilical vein endothelial cells (HUVECs) were purchased from the Cell Bank of Type Culture Collection of the Chinese Academy of Sciences (Shanghai, China). Endothelin-1(ET-1) and nitric oxide (NO) kits were purchased from Nanjing Jiancheng Bioengineering Institute (Nanjing, China). Alcalase, Dulbecco’s modified eagle’s medium (DMEM), norepinephrine (NE), neutrase, captopril (Cap), 3-(4,5-Dimethylthiazol-2yl)-2,5-dip-henyltetrazolium bromide (MTT), fetal bovine serum (FBS) and trifluoroacetic acid (TFA) were purchased from Beijing Solarbio Science & Technology Co., Ltd. (Beijing, China). ACEi peptides (purity > 95%) of SEGPK (MHP6), FDGPY (MHP7) and SPGPW (MHP9) were synthesized in Shanghai Apeptide Co., Ltd. (Shanghai, China).

### 3.2. Extraction of Collagens from Monkfish Swim Bladders

The swim bladder collagens (ASC-M and PSC-M) of monkfish were extracted according to the previous method [11]. Noncollagenous proteins were cleared up from monkfish swim bladders using 0.15 M NaOH at a sample/solution ratio of 1:12 (*w*/*v*) for 18 h at 4 °C. The NaOH solution was replaced every 3 h. After that, swim bladders were washed with distilled water (DW) and defatted with normal butanol (15%) with a sample/solution ratio of 1:18 for 32 h, and the solution was replaced every 8 h. Defatted monkfish swim bladders were washed three times with a 10-fold volume of cold DW.

Defatted swim bladder powder was dispersed in 0.5 M acetic acid at a swim bladder/solvent ratio of 1:15 (*w*/*v*) and stirred for extraction at 4 °C. After 48 h, the mixture was filtered with cheesecloth, and NaCl with a final concentration of 2.5 M was added into the filtered solution to precipitate collagen. The precipitate was collected via centrifugation at 16,000× *g* (TGL-16G, Shanghai, China) for 25 min at 4 °C. The solid precipitate was dialyzed with a 12-fold volume of acetic acid solution (0.1 M) at 4 °C. After 12 h, the dialysate was further dialyzed with a 20-fold volume of DW for 42 h, with the DW replaced every 6 h. The prepared dialysate was freeze-dried and named ASC-M.

The solid residue produced from the ASC-M preparation was subsequently mixed in a 10-fold volume of acetic acid solution (0.5 M). After the mixture was thoroughly stirred, porcine pepsin was added in the solution according to the dose of 20 U/g residues. The mixtures were sustained and stirred for extraction at 4 °C. After 48 h, PSC-M was prepared using the same preparation method as ASC-M. The yields of ASC-M and PSC-M were calculated based on the weight of the collagen extract according to the percentage of the total weight of the dried swim bladders.

### 3.3. Characterization of ASC-M and PSC-M

#### 3.3.1. Proximate and Amino Acid Analysis

The protein content of the swim bladders, ASC-M and PSC-M was determined by employing the Kjeldahl method. The fat, ash and moisture content in monkfish (*L*. *litulon*) swim bladders, ASC-M and PSC-M was determined using the AOAC method, with the numbers 960.39 (a), 950.46B and 920.153, respectively. Amino acid composition of ASC-M, PSC-M and CSC was measured according to the previous method [76].

#### 3.3.2. SDS-PAGE Patterns of ASC-M and PSC-M

SDS-PAGE patterns of ASC-M, PSC-M and CSC were determined using the previous method [77]. In brief, a 7.5% resolving gel and 4% stacking gel were employed in the electrophoresis experiment, and a high-MW protein marker was applied to determine the MWs of proteins.

#### 3.3.3. UV Absorption Analysis

A UV-1800 spectrophotometer was employed to record the UV adsorption spectrums of ASC-M, PSC-M and CSC from 200 to 400 nm. The ASC-M, PSC-M and CSC were separately dissolved in 0.5 M acetic acid solution with a collagen/solution ratio of 1:1000 (*w*/*v*).

### 3.4. Preparation of Hydrolysates of ASC-M and PSC-M

The preparation of a hydrolysate of PSC-M was carried out in accordance with the previous method [31,38]. The dispersions (1%, *w*/*v*) of ASC-M and PSC-M were separately degraded with alcalase (55 °C, pH 8.5, 4 h), neutrase (55 °C, pH 7.0, 4 H) and a double-enzyme system (alcalase (2 h) + neutrase (2 h)). The enzyme dose was designed as 2% (*w*/*w*). After the hydrolysis reaction, the proteases in the hydrolysate solution were inactivated in boiling water for 10 min. The prepared hydrolysates were centrifuged at 9000× *g* for 25 min, and the supernatants were freeze-dried and their ACEi ability was detected. The hydrolysate of PSC-M generated via the double-enzyme system displayed the highest ACEi ability value and was named PSC-MH.

### 3.5. Preparation of ACEi Peptides from PSC-MH

PSC-MH (100.0 mg/mL) was ultrafiltered with 1, 3.5 and 5 kDa MW cutoff membranes, and four peptide components, namely MH-I (MW < 1 kDa), MH-II (1 kDa < MW < 3.5 kDa), MH-III (3.5 kDa < MW < 5 kDa) and MH-IV (MW > 5 kDa), were prepared.

MH-I (5.0 mL, 60.0 mg/mL) was separated using a gel permeation chromatography column of Sephadex G-25 (2.6 × 150 cm). DW with a flow rate of 1.0 mL/min was used as the moving phase. The UV absorption of each collected eluate (3.0 mL) at 220 nm was analyzed. Lastly, three subfractions (MH-Ia, MH-Ib and MH-Ic) were isolated from MH-I.

Finally, MH-Ib (25 μL, 80.0 μg/mL) was purified with a Zorbax 300SB-C18 column (9.4 × 250 mm, 5 μm) in the RP-HPLC system. The column was eluted by a linear gradient of acetonitrile (containing 0.05% TFA) at 1.0 mL/min. The acetonitrile content was increased from 10% to 50% in 30 min. The UV absorption of the eluate at 220 nm was analyzed and twelve ACEi peptides (MHP1 to MHP12) were purified from MH-Ib, and their ACEi activity was measured.

### 3.6. Determination of ACEi Activity

The ACEi activity of PSC-MH, fractions and peptides (MHP1-MHP12) was determined by using FAPGG as a substrate [44]. Briefly, the initial volume was made up of a 50 µL volume of FAPGG (3 mM), ACE (1.25 mU) and the sample solution, respectively. These solutions were first preheated at 37 °C for 0.5 h for ash, and mixed and incubated for an additional 0.5 h. After that, 150 µL of glacial acetic acid was added to the solution to inactivate ACE. After that, the content of hippuric acid (HA) in the reaction mixture produced from the substrate reactions catalyzed by ACE was determined using HPLC at 228 nm. In short, using an isocratic system (pH 3.0) made up of 12.5% (*v*/*v*) acetonitrile in deionized water, the amount of free HA was evaluated using an HPLC system (Agilent 1200, Agilent Ltd., Santa Clara, CA, USA) on a Zorbax SB C-18 column (4.6 × 250 mm, 5 µm). The sample (10 µL) was eluted at a flow rate of 1.0 mL/min, and the absorbance at 228 nm was monitored to obtain the measurement [44]. A total of 50 µL of PBS buffer, used as the sample substitute, was added into the control reaction mixture. ACEi activity was calculated as follows:

ACEi activity (%) = [(HA _control_ − HA _sample_)/HA _control_] × 100% (1)

### 3.7. Sequence Identification of MHP6, MHP7 and MHP9

The amino acid sequences of MHP6, MHP7 and MHP9 were determined using a protein sequencer (Applied Biosystems 494, Perkin Elmer Co. Ltd. Foster City, CA, USA). The MWs of MHP6, MHP7 and MHP9 were measured using an ESI-Q-TOF-MS (Micromass, Waters, Milford, MA, USA) [78].

### 3.8. Molecular Docking Experiments on MHP6, MHP7 and MHP9

Molecular docking experiments were carried out according to the previous method [31]. The position and size of the binding pocket was confirmed with Chimera software (UCSF Chimera-1.15, San Francisco, CA, USA) through analyzing the interaction of the ACEi peptide (MHP6, MHP7 or MHP9) and ACE. Molecular docking and free energy calculation were carried out with the Autodock Vina. The best-ranked docking poses of MHP6, MHP7 and MHP9 in ACE were captured in accordance with the binding-energy scores.

### 3.9. Effects of MHP6, MHP7 and MHP9 on HUVECs

HUVECs were cultured in DMEM at 37 °C in a humidified 5% CO_2_ atmosphere, and the DMEM contained penicillin (100 U/mL), FBS (10%, *v*/*v*) and streptomycin (100 g/mL). After 24 h, the cells were used for follow-up experiments.

The MTT method was employed to detect the viability of HUVECs incubated with MHP6, MHP7 and MHP9 [38]. In brief, HUVECs were seeded in 96-well plates and cultured for 24 h. Subsequently, HUVECs were separately treated with MHP6, MHP7 and MHP9 (100, 200, 300 or 400 μM) at 37 °C. After 24 h, MTT was added into the cell culture to reach a final concentration of 2 mg/mL. After 4 h, DMSO was added into each well and the absorbance at 490 nm was determined. Cell viability was calculated according to the following formula:

Cell viability (%) = (A_sample_/A_control_) × 100 (2)

After treatment with ACEi peptides (MHP6, MHP7 and MHP9, respectively) for 24 h, the NO and ET-1 content in the HUVECs was separately determined according to their assay kits as per manufacturers’ protocol.

### 3.10. Statistical Analysis

Statistical analysis was carried out using SPSS 19.0 software. Experimental data were expressed as the mean ± standard deviations (SDs, *n* = 3) and were statistically analyzed using an ANOVA test. Duncan’s multiple range test was used to analyze the significant differences in each group (*p* < 0.05, 0.01 or 0.001).

## 4. Conclusions

In this study, collagens (ASC-M and PSC-M) were extracted from monkfish (*L. litulon*) swim bladders using acid and enzymatic methods. Their physicochemical properties indicated that ASC-M and PSC-M are similar to type I collagen. Moreover, three peptides with significant ACEi ability were separated from PSC-MH generated via a double-enzyme system and identified as SEGPK, FDGPY and SPGPW, respectively. SEGPK, FDGPY and SPGPW displayed remarkable hypotensive effects through their ACEi activity and through controlling NO and ET-1 generation in HUVECs. The ACEi activity of SEGPK, FDGPY and SPGPW was closely related to the interaction with the ACE’s active sites/pockets. Therefore, this work not only provides effective technology for the manufacture of collagens and novel ACEi peptides from monkfish swim bladders, but also helps to solve the pollution problem induced by fish by-products. More importantly, SEGPK, FDGPY and SPGPW could serve as safe functional substances for manufacturing significant blood pressure-lowering products, thereby controlling hypertension and CVDs.

## Figures and Tables

**Table 1:** Chemical compositions of swim bladders, ASC-M and PSC-M from monkfish (*L. litulon*).

Sample	Proximate Compositions (% Dry Weight)				Yield on Dry Weight Basis (%)
	Moisture	Protein	Fat	Ash	
Swim bladders	76.95 ± 3.68 ^a^	20.06 ± 1.09 ^a^	1.71 ± 0.14 ^a^	0.32 ± 0.03 ^a^	
ASC-M	4.51 ± 0.19 ^b^	93.68 ± 3.51 ^b^	0.37 ± 0.05 ^b^	1.02 ± 0.34 ^b^	4.27 ± 0.22
PSC-M	4.49 ± 0.35 ^b^	95.87 ± 2.89 ^b^	0.34 ± 0.03 ^b^	0.74 ± 0.26 ^c^	9.54 ± 0.51

^a–c^ Values with different letters indicate significant difference (*p* < 0.05).

**Table 2:** Amino acid compositions of ASC-M and PSC-M from swim bladders of monkfish (*L. litulon*) (residues/1000 residues).

Amino Acid	ASC-M	PSC-M	Collagen from Calf Skins (CSC)
Hydroxyproline	87.3	85.7	95.1
Aspartic acid/asparagine	45.9	43.8	45.7
Threonine	23.6	24.9	18.4
Glycine	325.2	314.9	330.6
Glutamine/glutamic acid	88.6	87.1	75.9
Proline	105.2	102.9	121.5
Serine	25.9	26.3	33.2
Isoleucine	16.6	15.1	11.4
Alanine	99.3	97.6	119.7
Cysteine	0	0	0.0
Valine	23.7	26.5	21.5
Methionine	5.8	4.5	6.1
Arginine	47.8	52.3	51.0
Leucine	32.5	29.4	23.4
Tyrosine	4.5	6.6	3.7
Hydroxylysine	8.2	10.9	7.7
Tryptophan	0.0	0.0	0.0
Lysine	32.6	38.5	26.5
Histidine	7.5	9.4	5.3
Phenylalanine	19.8	23.6	3.3
Total	1000.0	1000.0	1000.0
Imino acid	192.5	188.6	216.6

**Table 3:** Amino acid sequences, MWs, ACEi activity and affinities with ACE of MHP6, MHP7 and MHP9 from PSC-MH.

	RT (min)	Amino Acid Sequence	Observed MW/Theoretical MW (Da)	ACEi Activity (IC_50_, mg/mL)	Affinity (kcal/mol)
MHP6	10.08	SEGPK	516.5/516.6	0.63	−7.3
MHP7	12.68	FDGPY	597.6/597.6	0.94	−10.9
MHP9	14.91	SPGPW	542.6/542.6	0.71	−9.4

## Data Availability

Data are contained within the article.

## Abbreviations

ACE, angiotensin-I-converting enzyme; ACEi, angiotensin-I-converting enzyme-inhibitory; ASC-M, acid-soluble collagen; PSC-M, pepsin-soluble collagen; CSC, collagen from calf skins; DW, distilled water; SDS-PAGE, sodium dodecyl sulphate–polyacrylamide gel electrophoresis; UV, ultraviolet; MHP6, Ser-Glu-Gly-Pro-Lys (SEGPK); MHP7, Phe-Asp-Gly-Pro-Tyr (FDGPY); MHP9, Ser-Pro-Gly-Pro-Trp (SPGPW); IC_50_, half-maximal inhibitory concentration; HUVECs, human umbilical vein endothelial cells; NO, nitric oxide; ET-1, endothelin-1; CVDs, cardiovascular diseases; WHO, World Health Organization; Ang I, angiotensin I; Ang II, angiotensin II; Cap, captopril; HFD, high-fat diet; NAFLD, nonalcoholic fatty liver disease; AMPK, AMP-activated protein kinase; Nrf2, NF-E2-related factor 2; MW, molecular weight; BPs, bioactive peptides; HepG2, human hepatocellular carcinoma cells; NLRP3, NOD-like receptor thermal protein domain associated protein 3; RP-HPLC, reversed-phase high-performance liquid chromatography; RT, retention time; TFA, trifluoroacetic acid; NE, norepinephrine; DMEM, Dulbecco’s modified eagle’s medium; MTT, 3-(4,5-Dimethylthiazol-2yl)-2,5-dip-henyltetrazolium bromide; FBS, fetal bovine serum; HA, hippuric acid; HPLC, high-performance liquid chromatography.
